# Enhanced enzyme activity and stability through immobilization of recombinant chitinase on sodium alginate-modified rice husk beads for efficient decolorization of synthetic dyes

**DOI:** 10.1186/s13036-025-00546-4

**Published:** 2025-08-25

**Authors:** Shaimaa A. Nour, Ebtehag A. E. Sakr, Heba Kandil

**Affiliations:** 1 Chemistry of Natural and Microbial Products Department, Pharmaceutical and Drug Industries research Institute, National Research Centre (NRC), 33 El-Behouth St, Dokki, Giza 12622 Egypt; 2 Botany Department, Faculty of Women for Arts, Science and Education, Ain Shams University, Cairo, Egypt; 3 Polymers and Pigments Department, Chemical Industries Research Institute, National Research Centre, Giza, Egypt

**Keywords:** Dye removal, Immobilization, *Serratia marcescens*, Recombinant chitinase A, Rice husk

## Abstract

**Background:**

The energy efficiency and environmental friendliness of recombinant chitinase A make it a promising candidate for industrial applications as a sustainable catalyst. For the first time, a very stable and an efficient biocatalyst was developed to decolorize synthetic dyes by immobilizing *Serratia marcescens* chitinase A (SmChiA) onto beads comprised of sodium alginate (SA) and modified rice husk powder (mRHP). The mRHP was produced by treating rice husk powder with citric acid, which was then combined with SA at three different concentrations (25, 50 and 100% of SA weight) and cross-linked with calcium chloride to form the beads. 1-ethyl-3-(3-dimethylaminopropyl) carbodiimide facilitates the formation of amide bonds that covalently bind SmChiA to the beads. The effectiveness of the synthesis and immobilization processes was confirmed using characterization methods (scanning electron microscopy, SEM and Fourier transform infrared spectroscopy, FTIR).

**Results:**

Beads with 50% mRHP and 1.75 UmL^− 1^ of enzyme solution achieved the highest immobilization after 5 h of activation. The immobilized SmChiA demonstrated superior pH, temperature, and storage stability in respect to its free relative. The K_m_ value was 3.33 mg/mL, while the V_max_ was 4.32 U/mg protein/min. Activation energy (Ea), denaturation (E_d_), half-lives (T_1/2_), and decimal reduction time (D-values) were evaluated for immobilized and free SmChiA. The immobilization of SmChiA increased its affinity for the substrates by around 2.12 to 2.18 times. Compared to free chitinase, immobilized chitinase demonstrated greater durability after 22 reuses, maintaining its full activity. This proved the suitability of SA-mRHP beads as a cross-linker for chitinase immobilization. Crystal violet, malachite green, safranin, and methylene blue were more effectively decolorized from aqueous solutions by the immobilized SmChiA at a contact period of 84-h, dosage of 2.625 U/1.5 g, and temperature of 30 ^◦^C. Using an immobilized biocatalyst, the biodegradation was also examined using UV, FTIR, and SEM-EDX. The results confirmed the dye degradation.

**Conclusion:**

A variety of dyes could be safely removed from the environment using our bioremediation procedures. To the best of our knowledge, no studies had been conducted on the application of immobilized chitinase for dye removal.

## Introduction

Clean technology is growing across all industries particularly textile dying operations require an array of energy, water, chemicals, and dyeing colors. Serious air and water pollution issues could result from this [1]. Worldwide, the textile industry generates millions of cubic meters of dye-polluted wastewater annually. Discharging wastewater polluted with dyes into natural water streams is detrimental to the environment and public health [2]. Interestingly, most synthetic dyes have good visibility even at low doses. Many synthetic dyes are resistant to physico-chemical breakdown, non-biodegradable, and incredibly durable. Their chemical structures are also complex. Therefore, cleaning wastewater containing dyes has become an important environmental concern [3, 4].

Many methods have been developed to treat wastewater that contains dyes such as membrane separation [5], nitrifying-enriched activated sludge-based degradation [6], physical adsorption [7], and advanced oxidation processes [8]. These techniques, however, are expensive, time-consuming, and might inevitably produce wastes.

The employment of different biocatalysts in green chemistry has emerged as an effective tool in recent decades for addressing global issues in the manufacturing of recyclable products [9]. Because enzyme-based biocatalysts can successfully induce certain changes without being consumed, they have attracted a lot of interest [10, 11]. Researchers have also thoroughly examined the use of microbial enzymes to degrade the azo dyes. Among the enzymes that degrade dyes are azoreductases, peroxidases, and laccases [12]. Conversely, it has never been reported that the chitinase enzyme may eliminate dyes.

A wide variety of microorganisms, plants, animals, and insects produce hydrolytic enzymes called chitinases, which catalyze the breakdown of chitin. Microbial chitinases are essential for maintaining the nitrogen and carbon balance of an ecosystem. They are making progress in the fields of medicine, agriculture, food, medications, and environmental management [13]. When the chitinase producing gene is expressed heterologously in *E. coli*, the extracellular chitinolytic activity is increased by the chitinase enzyme SmChiA (GenBank: OR643436), which was obtained from *S. marcescens* (GenBank: OR793165). Shrimp waste produces chitooligosaccharides, which it breaks down very well [14]. Recombinant *S. marcescens* has demonstrated a markedly higher chitinase A (ChiA) efficiency in comparison to the wild strain [14]. The recombinant chitinase from *S. marcescens* GBS19 shows a lot of promise as a comprehensive pest management tool [15], and etc. However, as there is no recovery or utility for free enzymes, the process is not commercially feasible. Since immobilized enzymes provide greater stability, reusability, and cost savings, they are advised as a remedy for these problems [16]. So, the goal of both academic and industrial research is the synthesis of new adsorbent materials with useful uses.

The development of task-specific biosorbents through enzyme immobilization on the surface of certain active materials has drawn interest in the fields of energy, environmental science, and water treatment [17]. Chitosan beads, polyacrylamide, polyvinyl alcohol, calcium-alginate beads, hydrophobic sol-gels, macroporous exchange resins, nano-porous silica gel, and magnetic materials are among the supports used for enzyme immobilization [18–21]. Of these, alginate beads are a non-toxic, inert, biocompatible, and economical material that has attracted a lot of interest for enzyme attachment [18, 19]. Alginate is a naturally occurring anionic polysaccharide made up of repeating units of α-L-guluronic acid and β-D-mannuronic acid residues, and it is obtained from brown seaweeds [22]. It is especially well-appropriate for enzyme immobilization because it can form gels at room temperature and with certain divalent cations, like calcium [19, 23, 24].

Entrapment methods were utilized to immobilize the enzymes on the alginate beads in most papers that used alginate to immobilize enzymes [20, 22, 25–27]. This method is thought to be simple, non-toxic, and inexpensive [20, 28], but because of enzyme leakage, it frequently results in a reduction in catalytic effectiveness throughout the course of subsequent hydrolytic cycles [19, 20, 24, 27, 29]. Numerous strategies have been used to address this problem, including covalently immobilizing enzymes onto alginate, entrapment of enzyme-immobilized nanoparticles in place of native enzymes, and altering the porosity of the alginate matrix with substances like polycations, natural and synthetic polymers, crosslinking agents, and inorganic compounds [30]. One effective technique for stabilizing multiple enzymes is the covalent binding of different enzymes to alginate beads [20]. In order to covalently immobilize enzymes onto alginate, the current study uses a chemical technique that involves 1-ethyl-3-(3-dimethylaminopropyl) carbodiimide (EDAC). This chemical immobilization technique offers a practical and economical way to remove dye from wastewater by maintaining high enzymatic activity while also providing a robust and stable enzyme attachment. Alginate beads were enhanced by incorporating rice husk powder (RHP), an inexpensive and readily available byproduct of rice milling [31]. The addition of carboxylic groups to RHP through citric acid modification increased the number of surface area and active sites available for chemical modifications, thereby improving the efficiency of enzyme binding.

To the best of our knowledge, this study is the first to immobilize the *Serratia marcescens* ChiA enzyme on sodium alginate-modified rice husk beads to test its ability to degrade synthetic dyes. The purpose of this work is to develop an effective and reusable biocatalyst system that enhances enzyme stability and activity under various environmental conditions, aiming to provide a sustainable solution for removing harmful dyes from wastewater. The shape and chemical structure of beads (functional groups) were characterized before and after immobilization using scanning electron microscopy (SEM) and Fourier transform infrared spectroscopy (FT-IR). The stability and activity of the immobilized enzyme were evaluated at different temperatures and pH values, and its kinetic properties were studied. Recombinant SmChiA, expressed in *E. coli*, was applied to remove various dyes, and dye degradation efficiency was assessed using UV-VIS spectroscopy, FTIR, and SEM-EDX analyses.

## Materials and methods

### Materials

Rice husk powder (RHP) with an average particle size of 300 μm was generously supplied by nearby rice mills in El Sharkia, Egypt. Citric acid (CA) was provided from Morgan Chemical IND. Co. (Egypt). 1-ethyl-3-(3-dimethylaminopropyl) carbodiimide (EDAC), calcium chloride (CaCl_2_), and sodium alginate (SA) were supplied by Sigma-Aldrich (German).

### Methods

#### Production of recombinant chitinase from *S. marcescens*NRC408 (SmChiA)

Following overexpression in *Escherichia coli* BL21, the SmChiA-producing *S. marcescens* NRC408 strain (accession number OR793165) utilized in the degradation of shrimp waste was purified as previously mentioned [14]. In brief, *Escherichia coli* BL21 carrying SmChiA was cultured in LB broth containing kanamycin (50 µg/mL) at 37 °C with vigorous shaking until the optical density at 600 nm was reached at 0.6. Then, the culture was supplemented with 0.2 mM final concentration of isopropyl β-D-1-thiogalactoside (IPTG) and incubated at 37 °C. Then, the crude enzyme was precipitated. The recombinant chitinase was used for the following studies.

#### Enzyme activity estimation

The release of p-nitrophenol, which results in a color shift to yellow, was used to assess the activity of the pure recombinant SmChiA [32]. It was observed using a spectrophotometer at an absorption wavelength of 410 nm. One µmol of p-nitrophenol was released every minute, which was regarded as one unit of enzyme activity. Additionally, the amount of protein in the clear supernatant was quantified for an accurate assessment of enzyme activity [33].

#### Enzyme carrier preparation

##### Modification of RHP by CA

RHP citrate was prepared [34]. Briefly, 5 g of RHP was mixed with a specific amount of CA (dissolved in minimal water) under continuous stirring until a homogeneous paste formed. The paste was dried in a petri dish at 60 °C for 2 h, then incubated at 120 °C for 12 h. After incubation, the mixture was diluted with distilled water, vacuum-filtered to separate the modified RHP (mRHP), thoroughly washed, and dried at 70 °C for 24 h.

##### Preparation of SA-mRHP beads

The SA solution was mixed with different concentrations of mRHP to synthesis SA-mRHP beads. Three different amounts of mRHP were added to a 2% (w/w) SA solution: 0.5, 1, and 1.5 g agitated for 3 h at room temperature till homogeneity. The mixtures were then introduced dropwise using a 0.5 mm gauge syringe (without the needle) into a 3% (w/v) CaCl₂ solution under continuous stirring at 100 rpm. Spherical gel beads formed and were allowed to solidify in the gelling solution for 12 h [35]. Afterward, the beads were filtered, thoroughly washed with distilled water to remove residual CaCl₂, and collected as SA-mRHP beads.

##### Activation of gel beads and enzyme immobilization

The enzyme was fixed to the beads using amide linkages [36]. This method involved mixing 155 g of EDAC with beads in a buffer solution. Before the enzyme was added, the mixture was sonicated for 2 h. The resulting mixture was whirled at room temperature for different amounts of time. The activity of the immobilized SmChiA was determined by weighing the beads after they had been filtered and washed 3 times with distilled water.

##### Calculation of immobilization yield

Using the following formula, the immobilization yield was determined:

1 $${\rm{Immobilization\:yield = I / }}\left( {{\rm{A - B}}} \right)$$

A, B, and I stand for the total amount of enzyme added (U/carrier), unbound enzyme (U/carrier), and immobilized enzyme (U/carrier), respectively.

#### Box-Behnken design (BBD)

The immobilization processes for the SA-mRHP beads were optimized using BBD. SA-mRHP beads with different percent mRHP concentrations (A), recombinant SmChiA units (B), and loading time (C) were the 3 factors examined in the design of the experiment. The BBD consisted of 17 experimental runs with 5 central points, each of which was evaluated at 3 different levels [37]. The relation between the variables and the activity of the immobilized enzyme was examined using the following formula:

2 $${\rm{Y = }}{{\rm{B}}_{\rm{0}}}{\rm{ + \Sigma Bi Xi + \Sigma BijXiXj + \Sigma Bii X}}{{\rm{i}}^{\rm{2}}}$$

Where, Y: is the predicted immobilized activity; β0, ßi, ßij and ßii are the intercept of the model, linear, cross product and quadratic coefficients, respectively where Xi and Xj are the coded levels of the variables under investigation.

The best conditions for the actual immobilization of recombinant SmChiA are produced by this methodology, which allows for an exact assessment of the factors influencing enzyme activity and immobilization.

#### Characterization of the modified SA beads

- To determine the chemical structure of the sample, an ATR-FTIR spectrophotometer (Bruker VERTEX 80, Germany) with a resolution of 4 cm^-1^ and a refractive index of 2.4 was employed.
- The surface morphologies of the formed beads were assessed using a SEM (Quanta 250 FEG-FEI Company, United States) before and after enzyme immobilization.

### Biochemical characterization of immobilized recombinant SmChiA

#### Impact of pH

To determine how pH affected the activity of enzyme, immobilized recombinant SmChiA was tested across a pH range of 4 to 7.0 (acetate buffer: pH 4 to 5.5 and phosphate buffer: pH 6 to 7.0). The optimal pH was determined via measuring the enzymatic activity at each pH level. Additionally, to evaluate the stability of the immobilized recombinant SmChiA, the enzyme was pre-incubated for up to 120 min at the selected optimal pH. The initial activity of the enzyme, which was considered to be 100% in the absence of pre-incubation, was compared to the residual activity.

#### Effect of temperature

The effect of temperature on the activity of immobilized recombinant SmChiA was investigated by incubating the enzyme at various temperatures ranging from 40 to 80 °C. An Arrhenius plot was employed to measure the enzyme activation energy (Ea), which graphs the natural Lin of the relative activity against the inverse of the temperature in Kelvin. Utilizing the following formula, the activation energy was determined.

3 $${\rm{Slope = - Ea/R}}$$

Where R is the gas constant (8.314 J/mol·K) [38].

The enzyme was pre-incubated at 60–70 °C for a number of incubation times up to 120 min (without substrate) to assess the thermal stability of the free and immobilized recombinant SmChiA. The pre-incubated enzyme activity, which was fixed at 100%, was compared to the residual enzymatic activity.

#### Thermo-kinetic and thermo-dynamic parameters

The following calculation was used to assess the thermostability properties of the enzyme [39]:

4 $${{\rm{T}}_{{\rm{1/2}}}}{\rm{ = ln}}\left( {\rm{2}} \right){\rm{/}}{{\rm{K}}_{\rm{d}}}$$

5 $${\rm{Decimal\:reduction\:time }}\left( {{\rm{D - value}}} \right){\rm{ = ln}}\left( {{\rm{10}}} \right){\rm{/ }}{{\rm{K}}_{\rm{d}}}$$

Here, T is the temperature (K), E_d_ is the denaturation activation energy (KJmol^− 1^), and K_d_ is the rate constant for thermal deactivation. R is the gas constant (8.3145 J/mol·K), Kb is the Boltzman constant (1.38 × 10^-23^ J/K), and h is the Planck constant (6.626 × 10^-34^ J·s).

#### Impact of substrate concentration

In order to investigate how substrate concentration affects enzyme performance, 4-Nitrophenyl N-acetyl-β-d-glucosamine concentrations ranging from 0.02 to 2.8 mg/mL were evaluated on the free and immobilized recombinant SmChiA. To ascertain the kinetic parameters, a Lineweaver-Burk plot was employed [40]. Using the following formula, the maximal activity (V_max_) and Michaelis-Menten constants (K_m_) were determined:

6 $${\rm{1/V = }}\left( {{\rm{1/}}{{\rm{V}}_{{\rm{max}}}}} \right){\rm{ + }}\left( {{{\rm{K}}_{\rm{m}}}{\rm{/}}{{\rm{V}}_{{\rm{max}}}}} \right){\rm{ }}\left( {{\rm{1/S}}} \right)$$

where V_max_ is the maximum activity, K_m_ is the Michaelis-Menten constant, V is the immobilized and free enzyme activity (U/g), and S is the concentration of 4-Nitrophenyl N-acetyl-β-d-glucosamine (mg/mL).

#### Enzyme reusability

Under optimal conditions multiple hydrolysis cycles of 4-Nitrophenyl N-acetyl-β-d-glucosamine were performed to assess the reusability of the immobilized recombinant SmChiA. The beads were removed, cleaned, and used in a new reaction after every cycle. The process is altered when p-nitrophenol is produced, which gives the mixture its yellow color [32]. For 4 months, the storage stability of the immobilized enzyme was also evaluated weekly. The beads were stored in distilled water at 4 °C, and their activity was measured on a regular basis to assess their long-term stability.

#### Dyes removal assay and their analysis

The efficacy of dye removal was tested using seven dyes. The trails were conducted by varying the enzyme dosage, including immobilized recombinant enzyme on SA-mRHP beads (0.437 U/0.25 g, 0.875 U/0.5 g, and 1.31 U/0.75 g), free recombinant enzyme (0.437 U/0.25 mL, 0.875 U/0.5 mL, and 1.31 U/0.75 mL), and enzyme-free SA-mRHP beads (0.25 g, 0.5 g, and 0.75 g). Additionally, the effects of contact duration (24, 48, 72, and 84 h) and process temperature (30 °C and 40 °C) were evaluated. Each experiment involved mixing the material (either free enzyme, enzyme-immobilized beads, or non-enzymatic beads) with 5 mL of a dye solution (10 mg/L) and shaking the mixture. Blank experiments were carried out without the substance to confirm the validity of dye removal. The samples were separated using a 0.45 μm membrane after dye removal. After that, a UV–Vis spectrophotometry was used to examine the remaining dye. Malachite green, methylene blue, safranin, phenol red, eosin, crystal violet, and naphthol blue black had absorption wavelengths of 624, 665, 519, 550, 525, 592, and 620, respectively. The adsorption rate (%) was calculated using the formula removal rate (%) = (AC-AS)/AC, where AC is the absorbance of the control solution (original dye solution) and AS is the absorbance of the experimental group.

The UV-VIS spectra of the most promising treated dyes were done in the 300–800 nm region. The control and immobilized recombinant enzyme-treated dyes were recorded using a Shimadzu UV-1800 UV spectrophotometer. Prior to analysis, the samples were filtered. To evaluate the functional contribution of the enzyme in the process, the FTIR spectra of recombinant SmChiA were examined both before and after dye removal. Additionally, SEM-EDX analysis was performed following dye removal.

### Statistical analysis

The BBD model was statistically analyzed using analysis of variance (ANOVA). Fisher F-test was applied to evaluate the significance of the model equation, the estimation of multiple coefficients for each variable indicated the proportion of variance explained by the model; quadratic models were visualized using contour plots and response surface curves; and the coefficient of determination R^2^ and adjusted R^2^ were used to evaluate the predictive accuracy of the polynomial model equation (Design-Expert software, version 11.0 Stat-Ease Inc., Minneapolis, USA). The experimental results are expressed using the mean values of the treated dyes and the standard deviations of the mean values. One-way analysis of variance on the data was utilized using SPSS 20.0 software. The level of significance for probabilities (P-values) less than 0.05 and 0.01 (*P < 0.05, **P < 0.01 or ***P < 0.001) were significant, highly significant, and very highly significant, respectively.

## Results and discussion

**Fig. 1:**
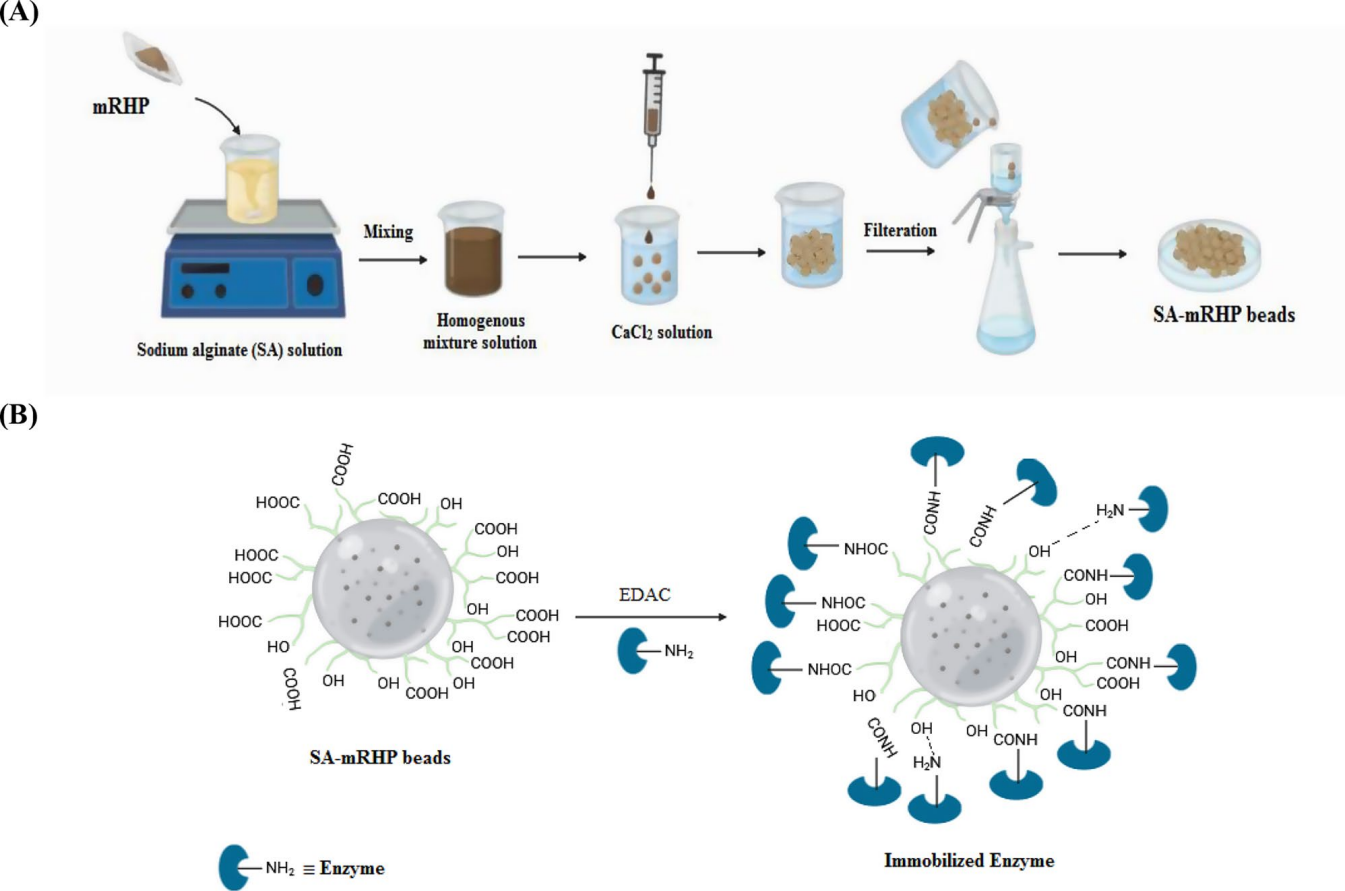
Schematic representation of the synthesis of SA-mRHP beads (**A**) and the immobilization of recombinant enzyme onto SA-mRHP beads **(B)**

The current investigation covalently immobilized recombinant SmChiA onto modified SA beads using a chemical method that exploited amide bonds assisted by EDAC (Fig. 1). This immobilization technique ensures a strong and stable enzyme attachment and high enzymatic activity while offering a cost-effective and efficient means of removing dye from solution. This change improves chemical stability and enzyme binding, but it also helps SA beads, which are prone to swell or crush in solutions with non-isotonic osmotic pressures. Therefore, the beads will be strengthened and more resistant to such conditions by the use of RHP. This improvement ensures the sustained functionality and long-term durability of the beads. The substantial surface area and active sites of RHP allow for further chemical modifications, enhancing its performance when integrated into SA beads. By modifying RHP with CA, carboxylic groups were introduced, improving enzyme binding and increasing the overall catalytic activity and immobilization efficiency of system. This approach contributed to the development of environmentally friendly and effective materials for dye treatment.

**Fig. 2:**
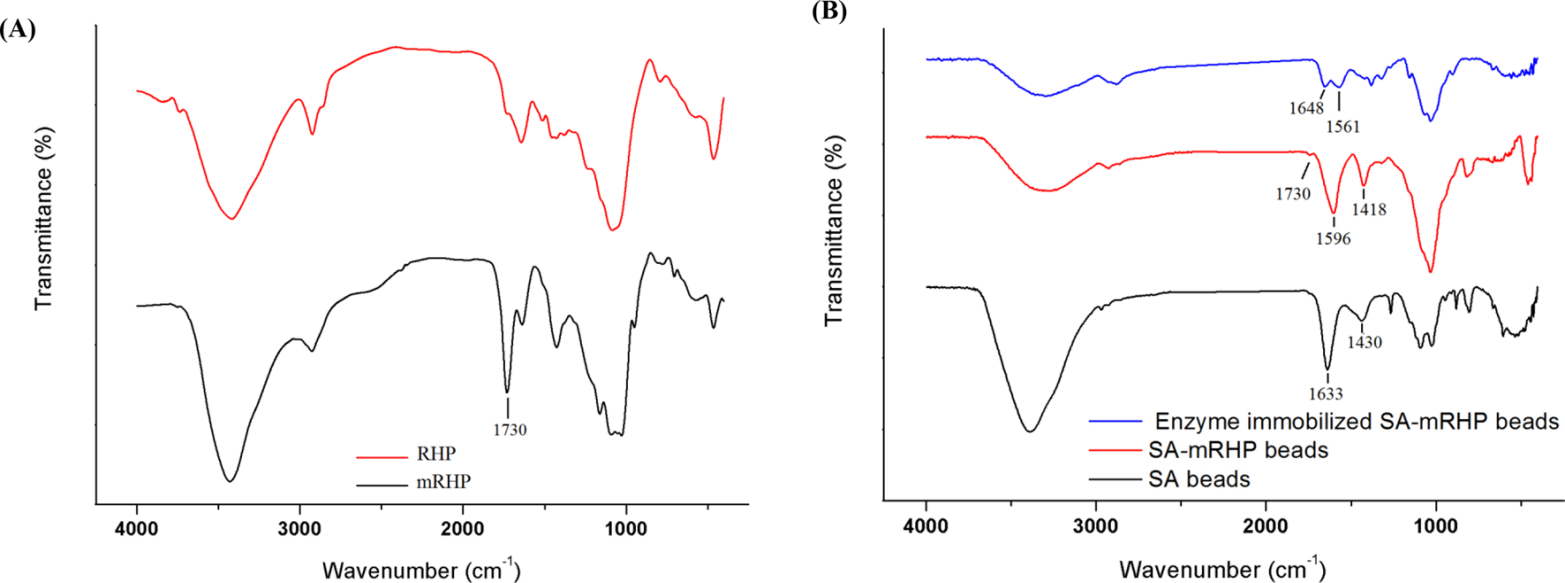
(**A**) FTIR of RHP and mRHP. (**B**) FTIR of SA beads, SA-mRHP beads before and after enzyme immobilization

FTIR spectra of mRHP and RHP were shown in Fig. 2. The stretching of the OH groups was identified as the cause of the broad band in the FTIR spectra of both samples, which were found to be positioned between 3165 and 3540 cm^− 1^. However, the strength of this band was larger in the newly modified sample than in the unmodified one. This was ascribed to the changed COOH symmetric stretching, indicating the successful grafting of CA onto the surface of RHP. Furthermore, a strong band at 1730 cm^− 1^ was found by comparing the spectra of RHP and mRHP, and this band was identified as belonging to the carboxylic acid (COOH) [41]. The successful addition of CA to the RHP surface was demonstrated by all of these indications.

Figure 2 showed the FTIR spectra of SA, SA-mRHP, and enzyme-immobilized SA-mRHP beads. According to the results, the characteristic FTIR bands of SA were observed in the spectra of all samples at 3385, 2963, 1633, 1430, and 1078 cm^-1^, which corresponded to the presence of O–H (stretching), C–H (blending), COO- (asymmetric stretching), COO- (symmetric stretching), and C-O-C (stretching) vibrations, respectively [42].

The incorporation of mRHP into SA resulted in distinct spectral changes. A new absorption band at 1730 cm^-1^ emerged, corresponding to the carboxyl (-COOH) group of mRHP [41]. Additionally, the mRHP had Si-O-Si and C-O stretching vibrations, typically observed between 1000 and 1100 cm^-1^ and 1065–1015 cm^-1^ [43], overlapped with the SA band at 1078 cm^-1^, leading to the formation of an intensified peak around 1000 cm^-1^. Furthermore, the COO^-^ bands of SA underwent a shift to lower frequencies, appearing at 1596 cm^-1^ (asymmetric stretching) and 1418 cm^-1^ (symmetric stretching). These spectral shifts suggest strong interactions between SA and mRHP, confirming the successful integration of mRHP within the SA matrix.

Upon enzyme immobilization, additional spectral modifications were observed. The symmetric stretching band of –COO^-^ and the –COOH band of mRHP was disappeared from SA, suggesting covalent binding of the enzyme through carboxylate groups. Moreover, the intensity of the asymmetric –COO^-^ stretching band at 1596 cm^-1^ decreased and shifted to 1561 cm^-1^ The formation of amide linkages was further confirmed by the emergence of a new band at 1648 cm^-1^, corresponding to the –C–N bond. All of these spectral shifts show that the enzyme was successfully immobilized onto SA-mRHP beads.

**Fig. 3:**
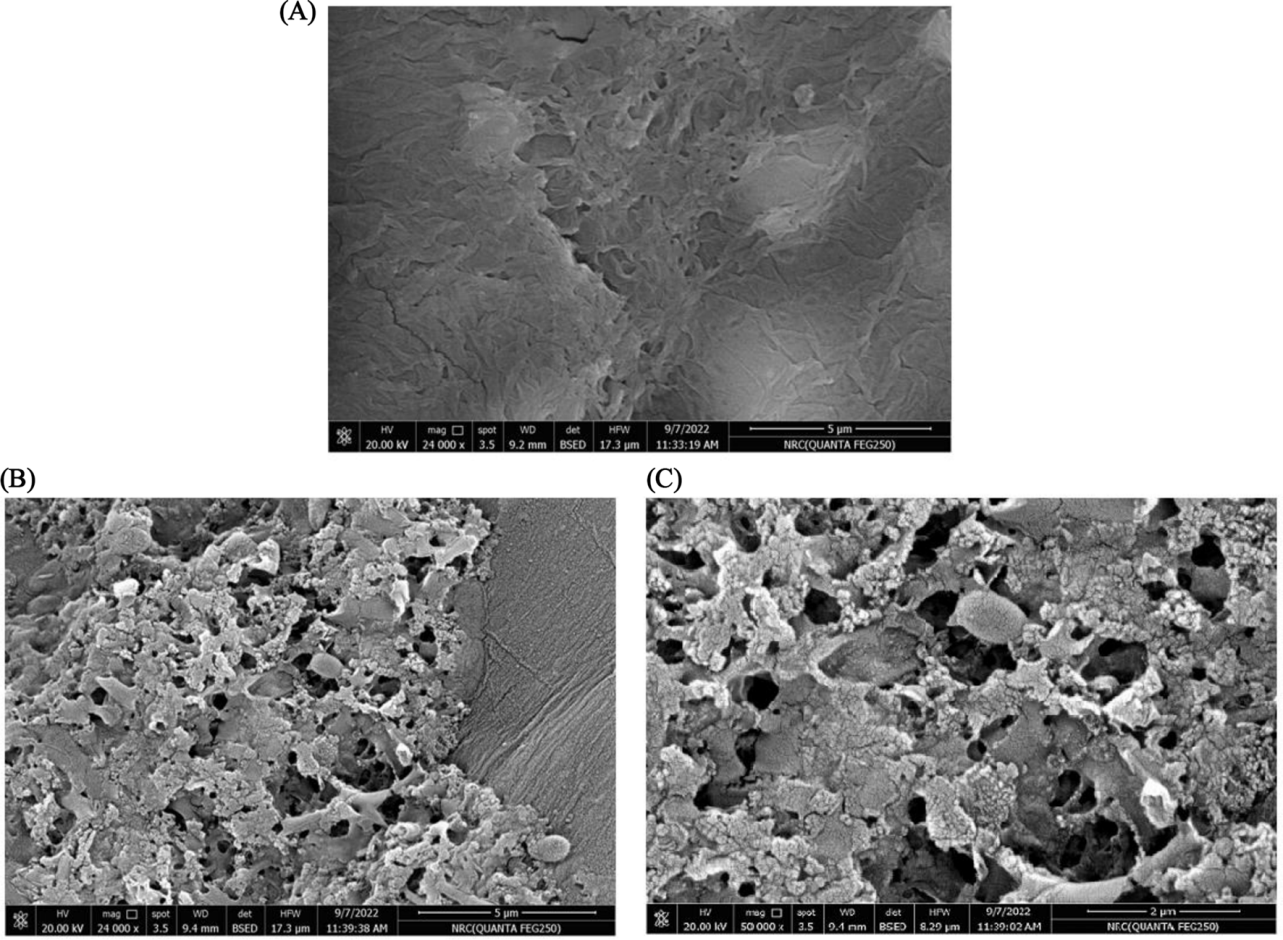
SEM images of SA beads: **(A)** before enzyme immobilization, and (**B**, **C**) after enzyme immobilization at low and high magnification, respectively

Furthermore, SEM was used to examine the surface morphology of SA beads before and after enzyme immobilization. A comparison of the SEM images of SA-mRHP beads before and after enzyme immobilization (Fig. 3) revealed noticeable changes in surface morphology. The SA-mRHP beads without enzyme displayed a relatively smooth and compact surface. This smooth texture is characteristic of the SA and RHP composite, which forms a uniform matrix with minimal surface irregularities. After enzyme immobilization, the SEM micrograph showed significant changes in surface morphology. The surface appeared rougher, with noticeable accumulations and irregular structures. These changes suggest the successful immobilization of the enzyme onto the bead surface, forming localized enzyme clusters. The rough and porous appearance may also result from the interaction between the enzyme and the SA-mRHP beads matrix.

### Enzyme activity

With an activity of 228.085 U/mL, the enzyme was successfully purified following the optimization of the recombinant SmChiA conditions [14]. The focus then shifted to immobilizing recombinant SmChiA on SA-mRHP beads in order to increase its reusability, which is essential for long-term commercial applicability.

After 5 h, the immobilization process loaded 2.25 U/mL onto 1.0 g of activated SA-mRHP beads with 100% of mRHP, yielding an estimated yield of 62.37 ± 1.14. With more optimization using a BBD, this outcome was significantly improved. Loading 1.75 U of SmChiA onto 1.0 g of activated SA-mRHP beads with 50% of mRHP and incubating them for 5 h produced a noteworthy immobilization yield of 96.45 ± 0.39 (Table 1).

**Table 1:** BBD for optimization of the immobilization process

Run	Beads conc. (rise husk concentration, X_1_)	Enzyme (U/g, X_2_)	Time (h, X_3_)	Immobilized yield (%)		Residual
				Actual value	Predicted value	
1	1(0)	2.25(0)	5(0)	62.37 ± 1.14	58.15	4.22
2	1(0)	2.25(0)	5(0)	62.01 ± 1.60	58.15	3.86
3	0.5(-)	1.75(-)	5(0)	96.45 ± 0.39	96.23	0.2175
4	1.5(+)	2.25(0)	6(+)	56.89 ± 0.14	57.41	-0.5150
5	1(0)	2.25(0)	5(0)	43.48 ± 1.32	58.15	-14.67
6	1.5(+)	1.75(-)	5(0)	69.38 ± 2.26	69.92	-0.5425
7	0.5(-)	2.25(0)	4(-)	93.44 ± 2.02	92.92	0.5150
8	1(0)	2.75(+)	4(-)	71.45 ± 2.82	72.51	-1.06
9	1(0)	2.25(0)	5(0)	61.34 ± 1.25	58.15	3.19
10	1(0)	2.25(0)	5(0)	61.55 ± 0.72	58.15	3.40
11	0.5(-)	2.25(0)	6(+)	76.85 ± 1.51	78.13	-1.28
12	1(0)	2.75(+)	6(+)	78.58 ± 1.41	77.85	0.7325
13	1.5(+)	2.75(+)	5(0)	95.62 ± 0.26	95.84	-0.2175
14	1.5(+)	2.25(0)	4(-)	95.33 ± 0.21	94.06	1.27
15	0.5(-)	2.75(+)	5(0)	89.66 ± 2.31	89.12	0.5425
16	1(0)	1.75(-)	4(-)	93.44 ± 0.41	94.17	-0.7325
17	1(0)	1.75(-)	6(+)	38.44 ± 1.97	37.38	1.06

The availability of functional groups, such as carboxyl and hydroxyl groups, that were available for covalent interaction with the enzyme and ensured stable immobilization is responsible for the higher immobilization effectiveness observed at 0.5 g mRHP concentration. On the other hand, the higher organic content at 1.0 and 1.5 g mRHP would have decreased the availability of functional groups, causing steric hindrance that prevented efficient enzyme attachment. Thus, 0.5 g mRHP created the perfect environment for robust covalent bonding, effective enzyme loading, and enhanced the mechanical robustness and immobilization efficiency of the beads, thus inhibiting hydrolysis and degradation [21]. Beads with 0.5 g mRHP were chosen based on this finding.

Statistical investigation revealed that the loading unit and polymer concentration had no obvious effect on the immobilization yield, while the loading period had a highly significant effect (*P* = 0.0007, < 0.05). According to multiple regression analysis, the model terms C, AB, BC, A^2^, and B^2^ were significant. P-values greater than 0.1000 were non-significant terms. The lack of fit F-value was 0.04 and indicated that the model lack of fit was not significant in respect to the pure error, with a 98.74% likelihood that noise was the source (Table 2).

**Table 2:** ANOVA analysis of BBD of the immobilization process

Source	df	Mean Square	F-value	*p*-value	
Model	9	586.88	14.81	0.0009	significant
Beads Conc. (rise husk conc.)	1	191.88	4.84	0.0637	
Enzyme (U/g).	1	176.72	4.46	0.0726	
Time (h)	1	1323.55	33.40	0.0007	
AB	1	272.75	6.88	0.0342	
AC	1	119.36	3.01	0.1263	
BC	1	965.03	24.35	0.0017	
A²	1	1665.53	42.02	0.0003	
B²	1	399.34	10.08	0.0156	
C²	1	28.22	0.7120	0.4267	
Residual	7	39.63			
Lack of Fit	3	2.59	0.0384	0.9885	not significant
Pure Error	4	67.41			
Cor Total	16				
Std. Dev.	6.30	**R²**	0.9501		
Mean	73.31	**Adjusted R²**	0.8859		
C.V. %	8.59	**Predicted R²**	0.9018		
Adeq. Precision	12.1884				

The R^2^ value of 0.9501, which accounted for 96.23% of the variation in the experimental findings, validated the validity of the second-order polynomial model used to predict the immobilization yield [44]. The predicted R^2^ (0.9018) and adjusted R^2^ (0.8859) agreed within a reasonable range, with a difference of less than 0.2. With noise having a 0.09% chance of generating high F-value, the strong significance of the model was indicated by F-value of 14.81. The results of the regression study yielded the second-order polynomial equation for estimating immobilization yield:

7 $$\eqalign{& {\rm{Y = 58}}{\rm{.15 - 4}}{\rm{.90}}{{\rm{X}}_{\rm{1}}}{\rm{ + 4}}{\rm{.70}}{{\rm{X}}_{\rm{2}}}{\rm{ - 12}}{\rm{.86}}{{\rm{X}}_{\rm{3}}}{\rm{ + 8}}{\rm{.26}}{{\rm{X}}_{\rm{1}}}{{\rm{X}}_{\rm{2}}} \cr & {\rm{ - 5}}{\rm{.46}}{{\rm{X}}_{\rm{1}}}{{\rm{X}}_{\rm{3}}}{\rm{ + 15}}{\rm{.53}}{{\rm{X}}_{\rm{2}}}{{\rm{X}}_{\rm{3}}}{\rm{ + 19}}{\rm{.89}}{{\rm{X}}_{\rm{1}}}^{\rm{2}}{\rm{ + 9}}{\rm{.74}}{{\rm{X}}_{\rm{2}}}^{\rm{2}}{\rm{ + 2}}{\rm{.59}}{{\rm{X}}_{\rm{3}}}^{\rm{2}} \cr} $$

**Fig. 4:**
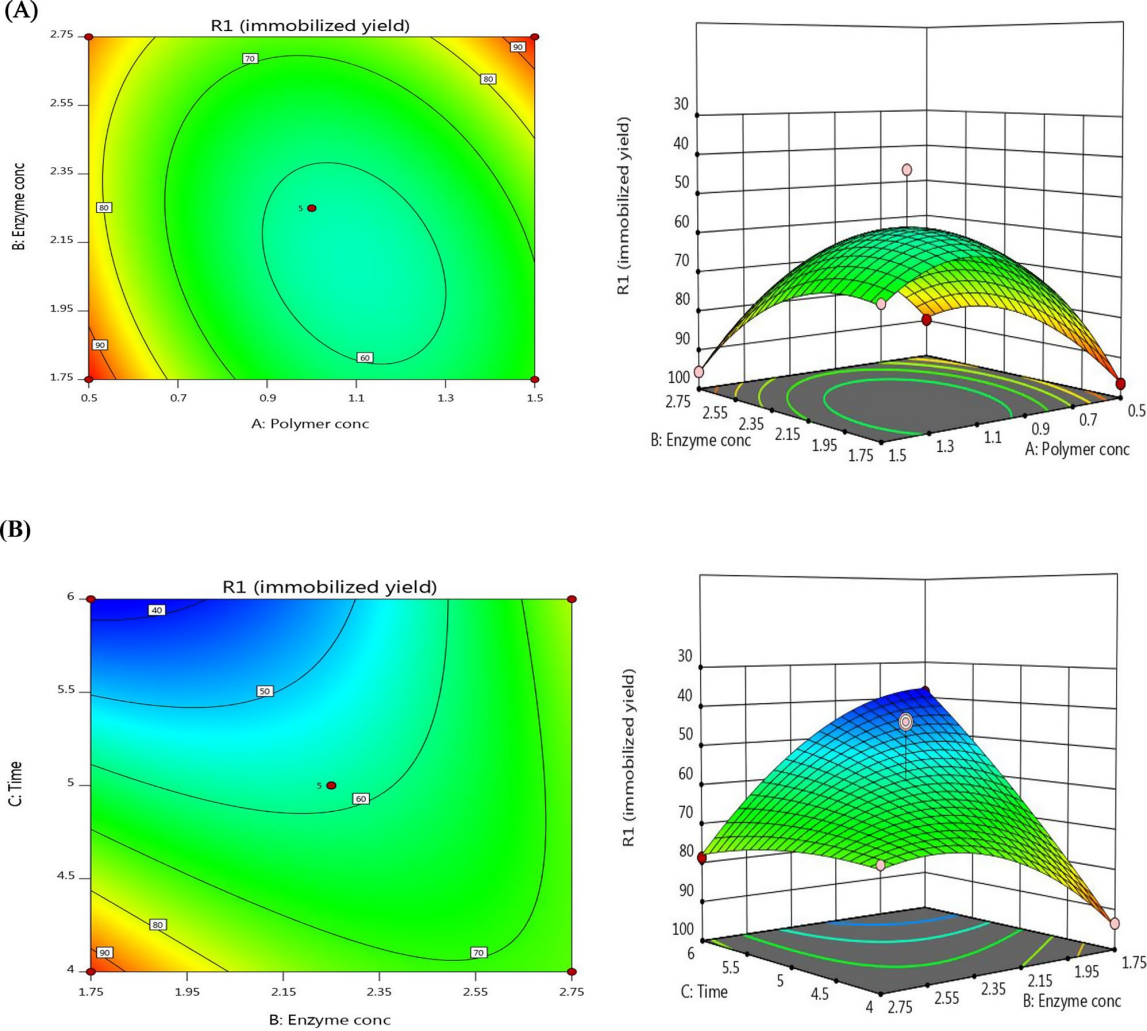
Response surface 3D and contour plots possessing the interactive effect between different variables on immobilized recombinant SmChiA; **(A)** Polymer concentration & Enzyme concentration **(B)** Enzyme concentration & Time

The contour and 3D plots illustrate how the variables combine to impact the productivity of enzymes (Fig. 4). Additionally, residual analysis showed a symmetrical distribution of residuals, confirming the correctness of model for most reported findings.

### Descriptions of recombinant SmChiA in the immobilized state

#### pH profile of immobilized SmChiA

**Fig. 5:**
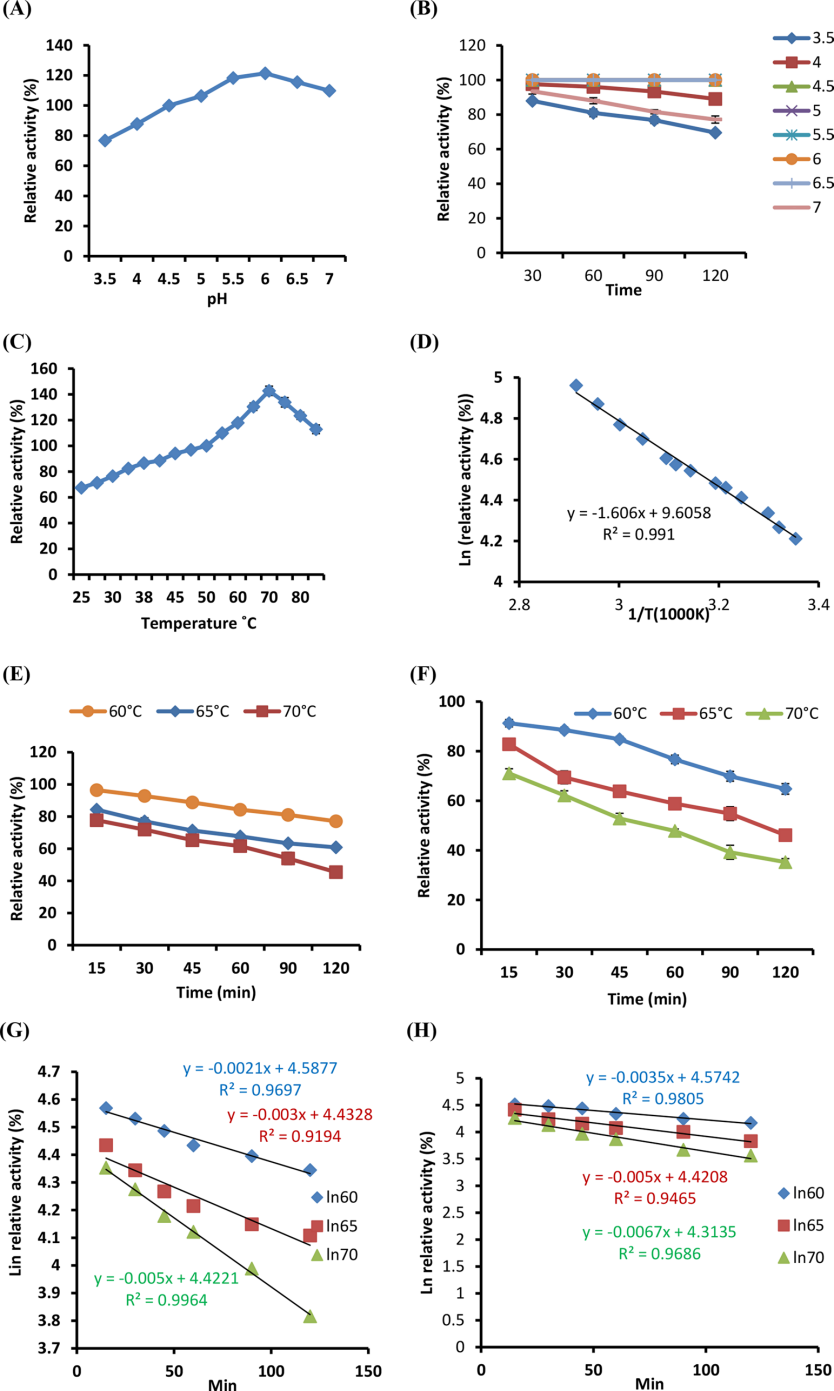

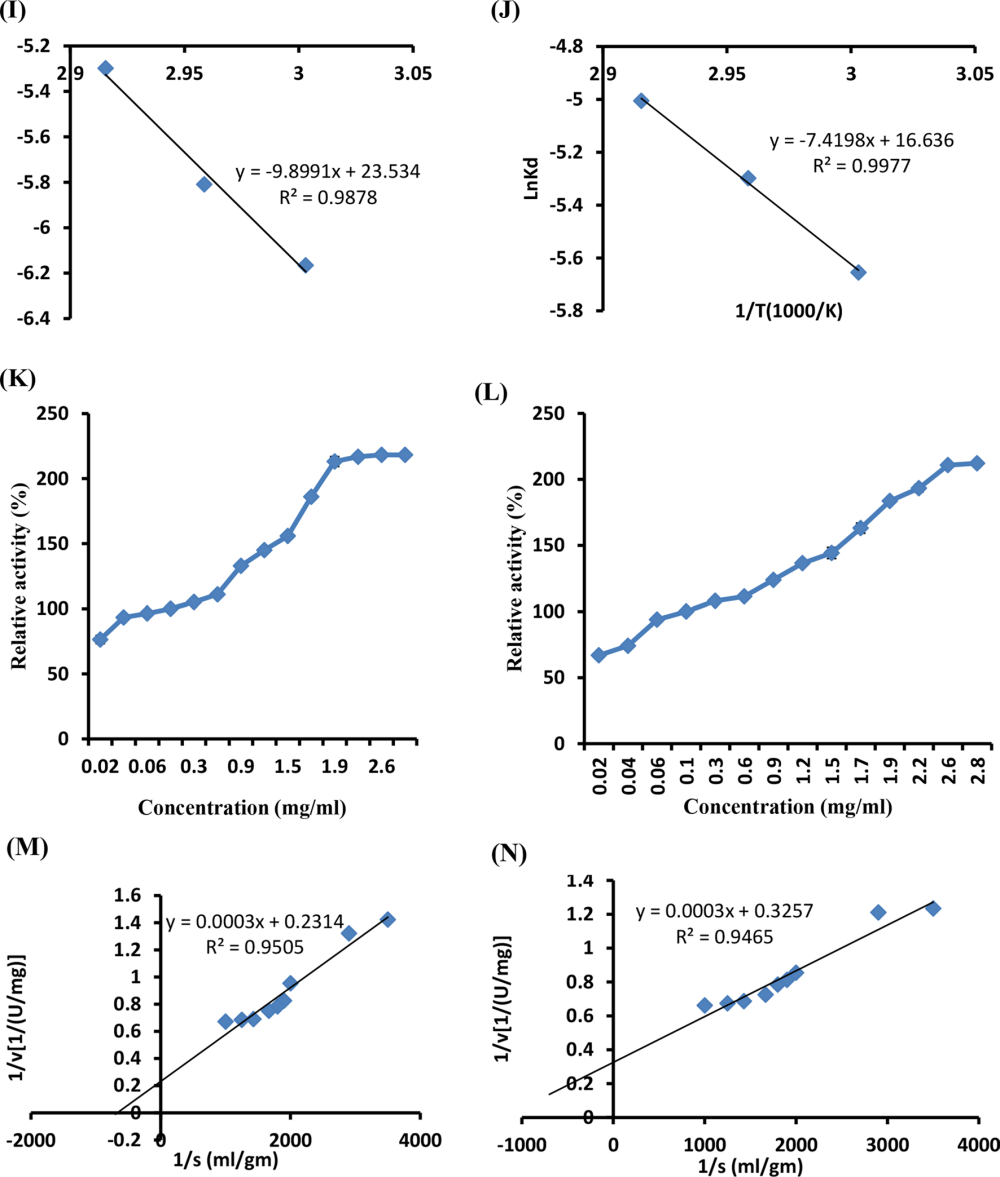
Effect of the reaction pH (control is pH 4.5) **(A)**, preincubation at different pH values for differenttime intervals (the activity of the immobilized recombinant enzyme without preincubation was considered 100% activity), on the activity of the immobilized enzyme **(B)**, the reaction temperature (50 °C is the control) **(C)**, the activation energy (Ea) **(D)**, and preincubation at different temperatures for different time intervals (the activity of the enzyme without preincubation was considered 100% activity) on the activity of the immobilized enzyme **(E)** and free enzyme **(F)**. First order of thermal deactivation of immobilized **(G)** and free **(H)**. Arrhenius plot to calculate activation energy for denaturation (Ed) immobilized **(I)** and free enzyme **(J)**. The activity of the immobilized enzyme using different substrate concentrations immobilized enzyme **(K)** and free enzyme **(L)**. Lineweaver–Burk plots were used to determine the values of the Michaelis–Menten constant (k_m_) and maximum reaction rate (V_max_) for both immobilized **(M)** and free SmChiA enzymes **(N)**

Using a 0.05 M phosphate buffer, the pH profile of the immobilized SmChiA was assessed. Following immobilization on SA-mRHP beads, the optimum pH slightly changed (Fig. 5A). Despite this change, the ideal pH of the immobilized enzyme remained at 6.0, which is closer to the optimal pH of 5.5 for free recombinant SmChiA [14]. The optimal pH for the activity of enzyme is altered during the immobilization process due to the covalent interaction between the enzyme and the supporting material. This indicates that variations in the charge distributions on active site residues typically cause a little shift in the optimal pH value [45, 46].

The immobilized SmChiA also demonstrated a high degree of retaining 100% of its enzymatic activity for 4.5–6.5 h (Fig. 5B). This high level of activity retention demonstrates the effectiveness of the immobilization process and suggests that the SA-mRHP beads provide an environment ideal for the long-term operation of the enzyme. Immobilized SmChiA on SA-mRHP beads is stable and functional due to the interaction between the carrier material and the enzyme structure. Due in significant part to the structural integrity of recombinant SmChiA and the composition and structural characteristics of SA-mRHP beads, enzyme activity is preserved following immobilization. These factors are required to ensure that the enzyme continues to catalyze within the desired pH range [45].

### Temperature profile of immobilized recombinant SmChiA

The temperature profile of immobilized SmChiA was evaluated in order to ascertain its thermal stability and optimal activity conditions. The 65 °C previously documented optimum temperature for the free enzyme [14], At 70 °C, the immobilized SmChiA on SA-mRHP beads showed the most effective catalytic activity. Because the free enzyme requires more activation energy than its immobilized form, the optimum temperature has changed. Immobilization frequently affects the structural flexibility and stability of the enzyme, which can alter the response of enzyme to heat. The immobilized SmChiA increased the ideal temperature which demonstrated its adaptation to its new environment within the gel matrix [46].

The enzyme activity of the immobilized recombinant SmChiA decreased after reaching the optimal temperature. As the temperature climbs above its stability threshold, the enzyme becomes more thermally denaturized, which is likely the source of this decrease in activity at higher temperatures (Fig. 5C). The results show that immobilization alters the thermal properties of enzyme and highlights how temperature controlled to preserve high enzymatic activity.

### Arrhenius plot of immobilized recombinant SmChiA

Using the Arrhenius equation, the activation energy (Ea) and catalytic efficiency of immobilized recombinant SmChiA were evaluated. The Arrhenius plot analysis demonstrated that the reaction had first-order kinetics and that the natural logarithm of residual activity and the reciprocal of temperature were linearly related. The activation energy was computed using this linearity from the gradient and intercept of the Arrhenius plot.

Arrhenius plot analysis revealed that the immobilized SmChiA had activation energy of 13.35 kJ/mol (Fig. 5D). When chitinase was immobilized on several carriers, its activation energy varied between 11.6 and 30.8 kJ mol^− 1^ [47]. The reduced activation energy for immobilized recombinant SmChiA indicates that less energy is required for the enzyme to reach its active site and combine with the substrate to form a complex.

The decrease in activation energy signifies that the catalytic efficiency of enzyme was optimized during the immobilization procedure, leading to greater efficacy at lower energy costs. This property is advantageous for industrial applications since it implies a decrease in overall manufacturing costs as a result of lower energy requirements. The enhanced stability and effectiveness of the immobilized SmChiA show promise for scalable and affordable industrial and medical processes [21, 48].

### Temperature stability of immobilized recombinant SmChiA

The temperature stability of the immobilized SmChiA was assessed by monitoring its activity over a 120-min during which the enzyme was incubated at various temperatures. At 40 °C, 45 °C, and 50 °C (data not shown), the immobilized SmChiA demonstrated excellent stability and sustained full enzymatic activity for the entirety of the 120-min incubation period (Fig. 5E). After 120 min, the activity of enzyme was still roughly 77.07% of what it had been at 60 °C. When the enzyme was immobilized, its activity decreased to 45.42% of its original value at 70 °C (Fig. 5E). According to Fig. 5F, the free enzyme only retained around 64.78 and 35.25% of its activity at 60 °C and 70 °C, respectively. The enzyme limited the structural changes carried from the immobilization process that could be the cause of its heat stability. Esawy et al. [49] also discovered outcomes.

The ability of the immobilized enzyme to sustain higher activity levels at higher temperatures suggests improved resilience, which is beneficial for applications in sectors where temperature changes are common for enzymes.

Based on the data acquired, a thermodynamic parameter was evaluated, including the E_d_, T_1/2_, and D-values (Fig. 3E, F). E_d_ values for the free and immobilized forms were 61.68 kJ mol^− 1^ and 82.3 kJ mol^− 1^ (Fig. I, J). At 60˚C, 65˚C, and 70˚C, the T_1/2_ of the immobilized and free forms was measured at 330, 231, and 138 min, respectively, and at 198, 138, and 103 min. According to Table 3, the D-values for the two enzyme types were 657, 460, and 343 min and 1096, 767, and 461 min, respectively.

**Table 3:** Thermodynamic parameters of free and immobilized SmChiA

	Immobilized SmChiA			Free SmChiA		
Temp	K_d_	T_1/2_	D-value	K_d_	T_1/2_	D-value
60	0.0021	330	1096	0.0035	198	657
65	0.003	231	767	0.005	138	460
70	0.005	138	461	0.0067	103	343

The expected T_1/2_ and D-values in our investigation were substantially higher than those in Mansour et al. [50] research on immobilized and free chitinase, which revealed that the T_1/2_ for the free and immobilized forms was 230, 138, 77 min and 345, 172, 115 min, respectively. The D-values for the two enzyme variants were 1150, 575, and 383 min and 766, 460, and 255 min, respectively.

### Effect of different substrate concentrations on immobilized recombinant SmChiA

The activity of both free and immobilized SmChiA was investigated in connection with varying p-nitrophenyl-b-D-N-acetyl glucoseaminide (PNP-b-GlcNAc) substrate concentrations. Both the free and immobilized enzymes exhibited their greatest activity at a substrate concentration of 2.8 mg/mL (Fig. 5K, L). Compared to the control (100%), the immobilized enzyme yielded somewhat higher activity than the free, 218% and 212%, respectively.

### Lineweaver–Burk plot

The K_m_ and V_max_ were examined. The K_m_ of both free and immobilized SmChiA enzymes was found to be 3.33 mg/mL. V_max_ for the free SmChiA enzyme was 3.07 U/mg protein/min. Conversely, the immobilized SmChiA enzyme displayed a higher V_max_ of 4.32 U/mg protein/min, suggesting that both the V_max_ and the overall catalytic efficiency are improved (Fig. 5M and N). This implies that the immobilized enzyme can achieve a greater maximal rate of reaction than its free counterpart. The K_m_ and V_max_ values, which are mostly influenced by the enzyme source, show how sensitive the enzyme is to the substrate; a drop in K_m_ and an increase in V_max_ indicate greater sensitivity to the substrate [51].

### Stability and operational conditions of immobilized recombinant SmChiA

Tests of stability and operational conditions of immobilized SmChiA were conducted using both long-term storage and recurrent use to determine its economic feasibility and reusability. Over time, the immobilized SmChiA showed outstanding stability. Over 22 cycles, the enzyme remained fully active. This finding is important from an economic standpoint since it lowers the total cost of using enzymes in industrial processes because they may be reused for many cycles. The usability and affordability of the immobilized enzyme in large-scale applications are influenced by its operational stability. After 16 reaction cycles, chitinase in fixed state maintained an impressive 39.7 ± 2.6% of its initial activity [46]. Furthermore, polyurethane/nano ZnO attached enzymes could be reused 10 times without undergoing a noticeable decrease in activity [52].

### Storage stability

The stability of the immobilized recombinant SmChiA was assessed for long-term storage at 4 °C. It was completely active for more than 38 d, but the free enzyme was only stable for 7 d before needing to be replaced because of contamination. The immobilized enzyme which sustain stability at lower temperatures highlights how well-suited it for long-term application in industrial settings. According to our results, the immobilized SmChiA shows strong operational stability and long storage capacity, which makes it ideal for a wide range of industrial applications. This stability promotes more cost-effective enzyme use in a variety of industrial processes in addition to improve the practical utility of enzyme.

### Utilization of free and immobilized recombinant SmChiA and SA-mRHP beads for dye removal

The catalytic efficiency of the chitinase enzyme may be altered by the immobilization procedure. The issue was investigated by comparing the decolorization capacity of chitinase immobilized onto SA-mRHP beads with that of the free enzyme and SA-mRHP beads using various synthetic dyes. This study examined the effects on material dosage, temperature, and contact time on the removal potential.

The immobilized recombinant SmChiA exhibited the maximum dye removal efficiency (%) when a dose of 1.31(U/0.75 g) was used and incubated for 84 h. The highest removal rates were observed for crystal violet (99.88 ± 0.64% at 30 °C), malachite green (98.54 ± 0.08% at 30 °C), safranin (92.01 ± 0.49% at 40 °C), and methylene blue (72.79 ± 0.15% at 30 °C) (Tables 4 and 5). These removal rates demonstrated the improved performance of the immobilized system and were noticeably greater than those attained by the free recombinant chitinase enzyme and enzyme-free beads. Both the capacity of immobilized enzyme to degrade dye molecules and the interaction of beads with the dye molecules are responsible for the observed increase in dye removal efficiency.

**Table 4:** Removal capability (%) of SmChiA immobilized onto SA-mRHP beads with 50% mRHP for structurally different synthetic dyes at 30 °C for different incubation times

Different dyes	SA-mRHP beads			Free SmChiA			Immobilized SmChiA		
	0.25 g	0.5 g	0.75 g	0.437(U/0.25 ml)	0.875(U/0.5 ml)	1.31(U/0.75 ml)	0.437(U/0.25 g)	0.875(U/0.5 g)	1.31(U/0.75 g)
**Dye removal (%)**									
Crystal violet									
24 h	13.19 ± 0.99^b^	17.90 ± 1.66^b^	27.08 ± 0.65^c^	24.72 ± 1.33^b^	28.96 ± 0.66^b^	36.72 ± 1.00^c^	75.95 ± 0.07^d^	75.60 ± 0.16^d^	89.64 ± 0.64^ab^
48 h	31.78 ± 1.33^a^	37.66 ± 0.33^a^	38.13 ± 0.98^b^	42.84 ± 0.33^a^	44.25 ± 4.32^a^	48.48 ± 0.99^b^	84.16 ± 0.17^c^	83.98 ± 0.03^c^	75.01 ± 0.34^b^
72 h	32.72 ± 0.67^a^	38.13 ± 0.99^a^	41.89 ± 0.90^a^	44.25 ± 0.33^a^	47.78 ± 0.65^a^	53.42 ± 0.67^a^	97.38 ± 0.03^b^	97.97 ± 0.07^b^	95.73 ± 0.64^a^
84 h	33.43 ± 0.33^a^	37.43 ± 0.66^a^	41.66 ± 1.30^a^	44.72 ± 0.32^a^	49.66 ± 4.65^a^	53.65 ± 0.33^a^	98.49 ± 0.20^a^	99.57 ± 0.06^a^	99.88 ± 0.64^a^
F-value	**67.32** ^***^	**182.52** ^***^	**92.14** ^***^	**353.45** ^***^	**17.14** ^*^	**197.84** ^***^	**12921.31** ^***^	**28114.86** ^***^	**5.905**
Malachite green									
24 h	10.37 ± 1.33^c^	16.98 ± 2.67^c^	25.47 ± 1.07^c^	19.81 ± 0.24^c^	31.13 ± 0.52^c^	35.84 ± 0.38^c^	44.15 ± 0.26^c^	61.22 ± 0.14^c^	61.98 ± 0.38^d^
48 h	18.86 ± 2.66^b^	27.35 ± 4.00^b^	36.79 ± 1.23^b^	27.35 ± 0.46^bc^	47.16 ± 0.83^b^	56.60 ± 0.21^b^	76.69 ± 0.13^b^	79.52 ± 0.40^b^	91.98 ± 0.45^c^
72 h	24.52 ± 2.61^ab^	39.62 ± 2.66^a^	47.16 ± 1.45^a^	36.79 ± 0.29^b^	58.49 ± 0.36^a^	67.92 ± 0.93^a^	85.75 ± 1.46^a^	92.92 ± 1.20^a^	95.47 ± 0.28^b^
84 h	30.18 ± 2.65^a^	40.283 ± 1.20^a^	53.77 ± 0.70^a^	50.00 ± 0.31^a^	59.43 ± 0.16^a^	69.81 ± 0.18^a^	85.28 ± 0.27^a^	94.15 ± 0.27^a^	98.54 ± 0.08^a^
F-value	**24.69** ^**^	**31.14** ^**^	**29.95** ^**^	27.19**	**26.04** ^**^	**68.33** ^**^	**1336.21** ^***^	1111.19***	**5734.32** ^***^
Safranin									
24 h	8.20 ± 0.90^c^	14.17 ± 0.91^c^	27.61 ± 0.19^b^	17.53 ± 0.73^d^	30.59 ± 0.7^c^	37.68 ± 0.66^c^	52.16 ± 0.42^c^	60.33 ± 0.58^d^	64.29 ± 0.11^d^
48 h	14.92 ± 0.54^b^	20.52 ± 0.24^b^	34.70 ± 0.15^a^	31.64 ± 0.18^c^	37.68 ± 0.66^b^	44.70 ± 0.15^b^	63.17 ± 0.16^b^	67.76 ± 0.12^c^	78.61 ± 0.26^c^
72 h	20.89 ± 0.55^a^	30.22 ± 0.39^a^	36.56 ± 0.72^a^	38.32 ± 0.09^b^	46.26 ± 0.87^a^	50.74 ± 0.63^a^	74.21 ± 0.64^a^	75.33 ± 0.51^b^	85.55 ± 0.15^b^
84 h	24.25 ± 0.37^a^	28.73 ± 0.13^a^	36.19 ± 0.04^a^	43.32 ± 0.09^a^	46.38 ± 0.06^a^	51.71 ± 0.64^a^	75.00 ± 0^a^	79.47 ± 0.76^a^	91.79 ± 0.12a
F-value	**57.21** ^***^	**27.31** ^**^	**50.267** ^**^	**806.11** ^***^	**77.34** ^***^	**41.21** ^**^	**120.96** ^***^	**440.98** ^***^	**241.63** ^***^
Methylene blue									
24 h	2.65 ± 0.02^c^	9.89 ± 0.40^d^	13.95 ± 0.76^d^	11.66 ± 0.08^c^	20.31 ± 0.81^c^	30.38 ± 0.87^c^	31.80 ± 0.21^d^	48.63 ± 0.96^d^	47.52 ± 0.65^d^
48 h	7.06 ± 0.71^b^	17.66 ± 0.78^c^	30.74 ± 0.20^c^	14.66 ± 0.43^c^	24.91 ± 0.17^b^	34.09 ± 0.89^b^	40.98 ± 0.94^c^	59.71 ± 0.73^c^	62.19 ± 0.08^c^
72 h	9.54 ± 0.06^b^	26.32 ± 0.51^b^	35.33 ± 0.57^b^	21.37 ± 0.81^b^	31.80 ± 0.21^a^	37.98 ± 0.59^a^	45.93 ± 0.64^b^	63.42 ± 0.76^b^	69.08 ± 0.13^b^
84 h	18.02 ± 0.12^a^	37.98 ± 0.59^a^	41.69 ± 0.61^a^	28.26 ± 0.86^a^	33.74 ± 0.56^a^	37.98 ± 0.74^a^	54.06 ± 0.36^a^	69.43 ± 0.46^a^	72.79 ± 0.15^a^
F-value	93.91^***^	134.89^***^	368.24^***^	41.26^**^	89.81^***^	63.04^***^	277.96^***^	443.82^***^	379.23^***^
Naphthol Blue Black									
24 h	2.80 ± 0.36^c^	4.84 ± 0.69^b^	7.65 ± 0.31^b^	11.54 ± 0.59^a^	13.54 ± 0.18^a^	14.05 ± 0.71^a^	10.45 ± 0.92^c^	12.21 ± 0.94^b^	12.50 ± 0^c^
48 h	5.35 ± 0.71^b^	7.14 ± 0.29^b^	9.18 ± 0.37^b^	12.57 ± 0.14^a^	13.39 ± 0.18^a^	15.07 ± 0.76^a^	9.18 ± 0.37^c^	13.52 ± 0.04^b^	21.68 ± 0.37^b^
72 h	8.67 ± 0.35^a^	11.98 ± 0.23^a^	14.03 ± 0.06^a^	14.00 ± 0.71^a^	15.03 ± 0.27^a^	17.28 ± 0.37^a^	16.58 ± 0.16^b^	22.19 ± 0.39^a^	32.65 ± 0.72^a^
84 h	9.18 ± 0.37^a^	11.98 ± 0.36^a^	14.54 ± 0.08^a^	15.48 ± 0.78^a^	15.34 ± 0.29^a^	17.33 ± 0.88^a^	20.91 ± 0.84^a^	24.23 ± 0.47^a^	33.92 ± 0.86^a^
F-value	**30.59** ^**^	**34.36** ^**^	**73.20** ^***^	**0.129**	**0.098**	**0.534**	**102.37** ^***^	99.62***	**417.13** ^***^
Eosin									
24 h	0	0.90 ± 0.36^b^	1.20 ± 0.48^c^	0.90 ± 0.36^b^	1.20 ± 0.48^c^	2.10 ± 0.84^c^	0.75 ± 0.30^c^	2.50 ± 0.97^c^	8.97 ± 0.59^c^
48 h	0.60 ± 0.24^ab^	0.90 ± 0.42^b^	1.80 ± 0.72^bc^	0.60 ± 0.24^b^	2.10 ± 0.84^c^	4.51 ± 0.81^c^	3.01 ± 0.20^bc^	7.83 ± 0.13^b^	11.44 ± 0.58^bc^
72 h	1.50 ± 0.6^ab^	0.60 ± 0.24^b^	3.61 ± 0.45^b^	6.02 ± 0.41^a^	6.62 ± 0.65^b^	9.03 ± 0.61^b^	6.62 ± 0.65^b^	9.33 ± 0.73^b^	14.15 ± 0.66^b^
84 h	3.31 ± 0.33^a^	5.72 ± 0.29^a^	6.32 ± 0.53^a^	5.42 ± 0.17^a^	12.04 ± 0.82^a^	14.75 ± 0.90^a^	13.25 ± 0.30^a^	18.07 ± 0.23^a^	21.98 ± 0.80^a^
F-value	**4.18**	11.63*	**17.92** ^**^	**17.44** ^**^	**29.43** ^**^	**59.41** ^***^	22.56**	**128.84** ^***^	**45.51** ^**^
Phenol red									
24 h	0.28 ± 0.09^b^	3.37 ± 0.08^a^	8.14 ± 0.61^b^	0.84 ± 0.27^c^	17.97 ± 0.75^a^	30.61 ± 0.80^a^	20.50 ± 0.56^c^	25.28 ± 0.09^c^	33.42 ± 0.70^c^
48 h	0	8.14 ± 0.61^a^	11.23 ± 0.6^ab^	7.86 ± 0.52^b^	13.76 ± 0.14^b^	24.71 ± 0.91^b^	24.43 ± 0.82^b^	37.19 ± 0.10^b^	41.79 ± 0.78^b^
72 h	2.80 ± 0.09^a^	11.51 ± 0.69^a^	14.60 ± 0.67^a^	10.95 ± 0.51^ab^	14.60 ± 0.67^b^	18.53 ± 0.93^c^	29.77 ± 0.53^a^	35.95 ± 0.51^b^	41.76 ± 0.97^b^
84 h	2.52 ± 0.81^a^	14.32 ± 0.58^a^	13.76 ± 0.4^a^	13.48 ± 0.31^a^	12.92 ± 0.13^b^	19.10 ± 0.11^c^	31.46 ± 0.07^a^	47.75 ± 0.28^a^	53.08 ± 0.99^a^
F-value	**7.81** ^*^	**1.699**	**9.30** ^*^	**19.43** ^**^	**7.54** ^*^	**24.91** ^**^	**42.53** ^**^	**118.35** ^***^	**123.58** ^***^

Results are expressed as mean ± standard deviations of values from triplicate experiments

* significant difference at *p* < 0.05

** significant difference at *p* < 0.01

***significant difference at *p* < 0.001

The values in the same column with the same letter for each dye are not significant different

SmChiA: chitinase A from *S. marcescens*

RHP: rice husk powder

**Table 5:** Removal capability **(%)** of SmChiA immobilized onto SA-mRHP beads with 50% mRHP for structurally different synthetic dyes at 40 °C for different incubation times

Different dyes	SA-mRHP beads			Free SmChiA			Immobilized SmChiA		
	0.25 g	0.5 g	0.75 g	0.437(U/0.25 ml)	0.875(U/0.5 ml)	1.31(U/0.75 ml)	0.437(U/0.25 g)	0.875(U/0.5 g)	1.31(U/0.75 g)
**Dye removal (%)**									
Crystal violet									
24 h	4.02 ± 3.99^b^	10.37 ± 1.69^c^	16.96 ± 0.98^c^	17.88 ± 2.31^b^	20.24 ± 0.31^c^	27.29 ± 2.31^b^	48.71 ± 2.66^c^	66.83 ± 0.20^c^	70.87 ± 0.07^d^
48 h	14.84 ± 1.33^a^	18.37 ± 0.99^b^	22.84 ± 0.56^b^	22.12 ± 1.65^b^	24.47 ± 0.36^b^	32.47 ± 0.35^a^	68.68 ± 0.37^b^	82.76 ± 3.01^b^	91.41 ± 0.03^c^
72 h	20.48 ± 0.63^a^	22.37 ± 0.60^a^	26.84 ± 0.99^a^	31.06 ± 0.31^a^	38.82 ± 1.97^a^	32.23 ± 0.68^a^	92.68 ± 0.10^a^	93.64 ± 0.19^a^	94.66 ± 0.17^b^
84 h	20.95 ± 0.66^a^	23.54 ± 1.66^a^	27.54 ± 0.63^a^	31.29 ± 1.30^a^	40.23 ± 1.30^a^	32.23 ± 0.01^a^	94.21 ± 0.07^a^	94.26 ± 0.13^a^	96.28 ± 0.13^a^
F-value	**26.69** ^**^	**40.69** ^**^	**65.53** ^***^	**36.11** ^**^	**139.08** ^***^	**8.56** ^*^	**519.13** ^***^	**143.50** ^***^	**21864.52** ^***^
Malachite green									
24 h	21.41 ± 0.73^b^	25.47 ± 0.39^c^	37.73 ± 0.23^c^	27.35 ± 0.41^c^	36.79 ± 0.71^c^	50.00 ± 0.29^b^	50.84 ± 0.91^b^	70.94 ± 0.24^c^	73.67 ± 0.92^c^
48 h	29.15 ± 0.54^a^	41.50 ± 0.63^b^	46.22 ± 0.16^b^	39.62 ± 0.35^b^	52.83 ± 0 ^b^	67.92 ± 0.45^a^	85.66 ± 0.04^a^	90.47 ± 0.17^a^	94.15 ± 0.09^ab^
72 h	30.66 ± 0.70^a^	51.88 ± 0.41^a^	62.26 ± 0.68^a^	49.05 ± 0.69^a^	58.49 ± 0.06^a^	70.75 ± 0.47^a^	85.94 ± 0.34^a^	89.43 ± 0.40^b^	93.58 ± 0.49^b^
84 h	30.84 ± 0.25^a^	54.24 ± 0.78^a^	62.73 ± 0.22^a^	50.94 ± 0.19^a^	60.37 ± 0.74^a^	69.81 ± 0.13^a^	85.94 ± 0.32^a^	90.00 ± 0^ab^	95.18 ± 0.87^a^
F-value	**15.9** ^*^	**54.09** ^**^	**42.04** ^**^	**20.94** ^**^	**57.29** ^**^	**16.66** ^*^	**36.81** ^**^	**1940.49** ^**^	**1143.17** ^***^
Safranin									
24 h	13.43 ± 0.28^c^	20.52 ± 0.24^c^	35.07 ± 0.46^c^	28.84 ± 0.33^c^	32.68 ± 0.66^d^	36.60 ± 0.45^d^	53.88 ± 0.06^d^	60.48 ± 0.51^d^	80.67 ± 0.16^c^
48 h	20.14 ± 0.93^b^	28.65 ± 0.67^b^	39.02 ± 0.69^b^	37.53 ± 0.73^b^	44.17 ± 0.91^c^	50.29 ± 0.85^b^	56.94 ± 0.03^c^	69.51 ± 0.49^c^	81.75 ± 0.37^c^
72 h	28.09 ± 0.71^a^	38.99 ± 0.25^a^	44.25 ± 0.37^a^	38.65 ± 0.67^b^	45.74 ± 0.63^b^	54.14 ± 0.18^a^	65.82 ± 0.09^b^	70.74 ± 0.63^b^	86.15 ± 0.67^b^
84 h	26.71 ± 0.64^a^	38.58 ± 0.21^a^	43.99 ± 0.25^a^	43.69 ± 0.40^a^	47.53 ± 0.13^a^	48.65 ± 0.67^c^	74.29 ± 0.26^a^	78.88 ± 0.06^a^	92.01 ± 0.49^a^
F-value	**38.67** ^**^	**184.72** ^***^	**30.49** ^**^	**207.99** ^***^	**1616.4** ^***^	**397.80** ^***^	**287.68** ^***^	**3017.61** ^***^	**213.28** ^***^
Methylene blue									
24 h	0.70 ± 0.67^d^	3.88 ± 0.69^d^	7.24 ± 0.38^d^	9.18 ± 0.73^c^	14.31 ± 0.1^d^	26.50 ± 0.18^c^	27.38 ± 0.52^c^	37.72 ± 0.08^d^	49.82 ± 0.33^c^
48 h	3.35 ± 0.69^c^	9.54 ± 0.06^c^	12.72 ± 0.08^c^	11.30 ± 0.74^c^	16.60 ± 0.78^c^	24.02 ± 0.83^c^	35.33 ± 0.57^b^	52.29 ± 0.68^c^	59.89 ± 0.4^b^
72 h	6.71 ± 0.38^b^	15.90 ± 0.11^b^	25.79 ± 0.51^b^	16.43 ± 0.11^b^	24.38 ± 0.16^b^	30.74 ± 0.2^b^	38.86 ± 0.93^ab^	55.83 ± 0.04^b^	62.01 ± 0.41^b^
84 h	13.78 ± 0.09^a^	19.61 ± 0.13^a^	31.44 ± 0.88^a^	30.63 ± 0.60^a^	31.09 ± 0.54^a^	35.15 ± 0.90^a^	41.87 ± 0.28^a^	60.07 ± 0.07^a^	67.31 ± 0.45^a^
F-value	163.98^***^	84.72^***^	329.87^***^	254.37^***^	227.74^***^	47.23^**^	23.41^**^	187.94^***^	134.76^***^
Naphthol Blue Black									
24 h	4.33 ± 0.67^c^	7.65 ± 0.31^b^	9.18 ± 0.37^c^	8.67 ± 0.35^b^	10.96 ± 0.94^b^	15.05 ± 0.21^c^	11.22 ± 0.45^c^	15.05 ± 0.51^c^	18.36 ± 0.73^c^
48 h	6.63 ± 0.27^b^	8.41 ± 0.84^b^	11.47 ± 0.96^b^	8.41 ± 0.84^b^	11.47 ± 0.96^b^	15.30 ± 0.32^c^	9.43 ± 0.88^c^	19.89 ± 0.80^b^	25.51 ± 0.02^b^
72 h	10.20 ± 0.41^a^	12.50 ± 0^a^	16.07 ± 0.14^a^	15.30 ± 0.61^a^	20.40 ± 0.82^a^	24.48 ± 0.98^a^	21.17 ± 0.35^b^	30.61 ± 0.22^a^	32.65 ± 0.31^a^
84 h	10.45 ± 0.92^a^	11.98 ± 0.36^a^	15.05 ± 0.11^a^	15.56 ± 0.12^a^	20.40 ± 0.72^a^	20.66 ± 0.33^b^	25.25 ± 0.51^a^	30.10 ± 0.20^a^	31.88 ± 0.78^a^
F-value	**29.77** ^**^	**24.77** ^**^	**89.28** ^***^	**54.07** ^**^	**57.68** ^***^	**70.51** ^***^	**92.40** ^***^	**74.36** ^***^	**47.643** ^**^
Eosin									
24 h	0	0	0.60 ± 0.24^b^	0	0	1.20 ± 0.48	0	0.30 ± 0.12^c^	0.30 ± 0.12^c^
48 h	1.50 ± 0.60^a^	0.30 ± 0.12^b^	0	1.80 ± 0.72	1.20 ± 0.48	3.61 ± 0.45	2.10 ± 0.84^b^	2.40 ± 0.96^b^	5.12 ± 0.05^b^
72 h	2.40 ± 0.96^a^	1.50 ± 0.6^b^	0	4.21 ± 0.69	7.22 ± 0.89	5.42 ± 0.17	6.02 ± 0.41^a^	10.54 ± 0.22^a^	11.74 ± 0.70^a^
84 h	2.10 ± 0.84^a^	5.12 ± 0.05^a^	6.62 ± 0.65^a^	1.20 ± 0.48	9.03 ± 0.61	9.63 ± 0.86	7.53 ± 0.01^a^	10.24 ± 0.10^a^	13.55 ± 0.42^a^
F-value	**1.689**	**22.15** ^**^	**57.33** ^***^	**0**	**0**	**0**	**13.96** ^*^	**123.33** ^***^	**137.22** ^***^
Phenol red									
24 h	1.12 ± 0.36^c^	6.46 ± 0.07^c^	13.48 ± 0.31^b^	26.68 ± 0.54^b^	28.93 ± 0.26^b^	33.42 ± 0.37^c^	40.16 ± 0.85^d^	47.19 ± 0.11^c^	51.82 ± 0.19^c^
48 h	7.30 ± 0.34^b^	12.07 ± 0.87^b^	23.87 ± 0.64^a^	29.21 ± 0.35^b^	35.39 ± 0.33^ab^	39.60 ± 0.67^b^	52.19 ± 0.31^c^	52.89 ± 0.33^b^	57.61 ± 0.24^b^
72 h	13.76 ± 0.40^a^	17.41 ± 0.57^a^	27.52 ± 0.81^a^	42.13 ± 0.48^a^	42.41 ± 0.57^a^	47.75 ± 0.28^a^	56.96 ± 0.63^b^	53.37 ± 0.08^b^	57.30 ± 0.34^b^
84 h	12.92 ± 0.13^a^	16.57 ± 0.30^a^	25.00 ± 0^a^	41.85 ± 0.39^a^	44.38 ± 0.2^a^	46.62 ± 0.92^a^	60.53 ± 0.37^a^	61.79 ± 0.78^a^	64.32 ± 0.58^a^
F-value	**23.79** ^**^	**55.69** ^**^	**24.85** ^**^	**62.74** ^***^	**7.19** ^*^	**30.52** ^**^	**146.96** ^***^	**161.47** ^***^	**51.57** ^**^

Results are expressed as mean ± standard deviations of values from triplicate experiments

* significant difference at *p* < 0.05

** significant difference at *p* < 0.01

***significant difference at *p* < 0.001

The values in the same column with the same letter for each dye are not significant different

SmChiA: chitinase A from *S. marcescens*

RHP: rice husk powder

In contrast to the other dyes, eosin exhibited much lower removal rates after 84 h (21.98 ± 0.80% at 30 °C and 13.55 ± 0.42% at 40 °C). This reduced removal effectiveness is probably caused by the negatively charged of eosin dye molecules and electrostatic repulsion with the carboxylate groups of alginate bead, which prevents the dye from interacting and attaching to the beads. However, since the enzyme catalyzes the disintegration of the dye molecules, its presence on the beads would aid in the dye removal.

Tables 4 and 5 also demonstrate that the removal efficiency rose from 24 to 84 h for most dyes, indicating that the removal effectiveness increased with contact time. This suggests that as dye molecules interacted with the material over time, both the enzyme activity and the bead absorption capacity increased.

With respect to temperature, Tables 4 and 5 showed the effectiveness of dye removal rose with increasing temperature with dyes such as phenol red and safranin. In particular, phenol red had the biggest improvement in removal at 40 °C, whereas safranin removal only little changed with temperature. This increase was due to its molecules that increased mobility at the higher temperature improving the enzyme-dye molecule interaction.

As shown in Tables 4 and 5, the effect of material dose revealed that all dyes showed greater removal effectiveness as the quantity of enzyme-immobilized adsorbent beads increased. A greater material dose results in more active sites and an increased enzyme load, which improves the adsorption of dye molecules as well as their enzymatic breakdown (decolorization), increasing the overall efficiency of dye removal. The immobilized enzyme and dye molecules interact more frequently as a result of the increased surface area and enzyme concentration, which improves dye removal efficiency. Based on these findings, it was determined that 1.31 U/0.75 g was the ideal concentration of immobilized chitinase for the maximum dye removal. The highest rates of removal for crystal violet, malachite green, safranin, and methylene blue were obtained at this concentration, which were used for further examination.

The results demonstrated that the range of chemical structures of dyes may contribute to differences in decolorization efficacy [53]. The causes for the increased dye removal with contact time are the first stage of adsorption, when empty surface sites are available, and the challenge of filling the remaining unfilled sites once equilibrium is attained [54]. The repelling forces between the basic dye molecules and the immobilized recombinant SmChiA are most likely the cause of this. The adsorption rate dropped from 72 to 84 h. Dye molecules occupying a large number of adsorption sites may have caused the adsorption rate of immobilized chitinase to gradually slow down. Furthermore, there is a steady decrease in both the dye concentration in the solution and the differential between the dye concentrations inside and outside the immobilized enzyme. The adsorption rate gradually drops as a result, a sign that there is insufficient energy to maintain the ability of immobilized enzyme to adsorb the dye molecules. The incredibly high uptake and removal efficiency of the dye molecules during their initial contact time with the adsorbent may be related to high dye–immobilized enzyme interactions because the immobilized chitinase surface has a large number of active absorbent sites and there is little interference from solute-solute interactions (Dougan et al. [55]).

Results indicated that contact periods exceeding 72 h had no discernible effect on the rate at which adsorbents removed certain dyes. Dye molecules agglomerate when contact time increases, potentially preventing deeper penetration into the higher-energy adsorbent active sites [56, 57]. With a longer contact time until equilibrium, dye adsorption increased, and the amount of dyes adsorbed at equilibrium indicated the maximum adsorption capacity of adsorbent under the specific working conditions [58]. Numerous studies have demonstrated that immobilized laccase has a higher capacity for dye decolorization and degradation as well as improved recyclability potential when compared to free enzyme [59, 60].

Table 5 shows that a few dyes can be removed more effectively when adsorbents are used at temperatures as high as 40 °C. The increase in surface activity is the main factor responsible for this improvement, demonstrating that the adsorption of immobilized chitinase enzyme up to 40 °C is an endothermic process within the designated temperature range [61, 62]. According to the findings, raising the temperature causes the dye ions to become more mobile, which allows the large dye molecule to penetrate farther [63]. As the temperature rises, the surface activity and kinetic energy of adsorbent particles may increase [64]. This higher temperature may also increase the frequency of collisions between adsorbents and dyes, which could result in enhanced adsorption [64, 65]. Furthermore, a higher temperature may increase the mobility and solubility of the dye molecules, strengthening their interactions with the solvent and accelerating the rate of diffusion [66, 67].

The amount of dye absorbed per unit of adsorbent weight falls as the adsorbent dosage increases, indicating a decrease in adsorption capability. In fact, adsorption capacity decreases due to increased adsorbent buildup and high active site availability. Therefore, dye removal is limited for constant dye concentrations and increasing adsorbent concentrations [68].

### The UV–Vis absorption

**Fig. 6:**
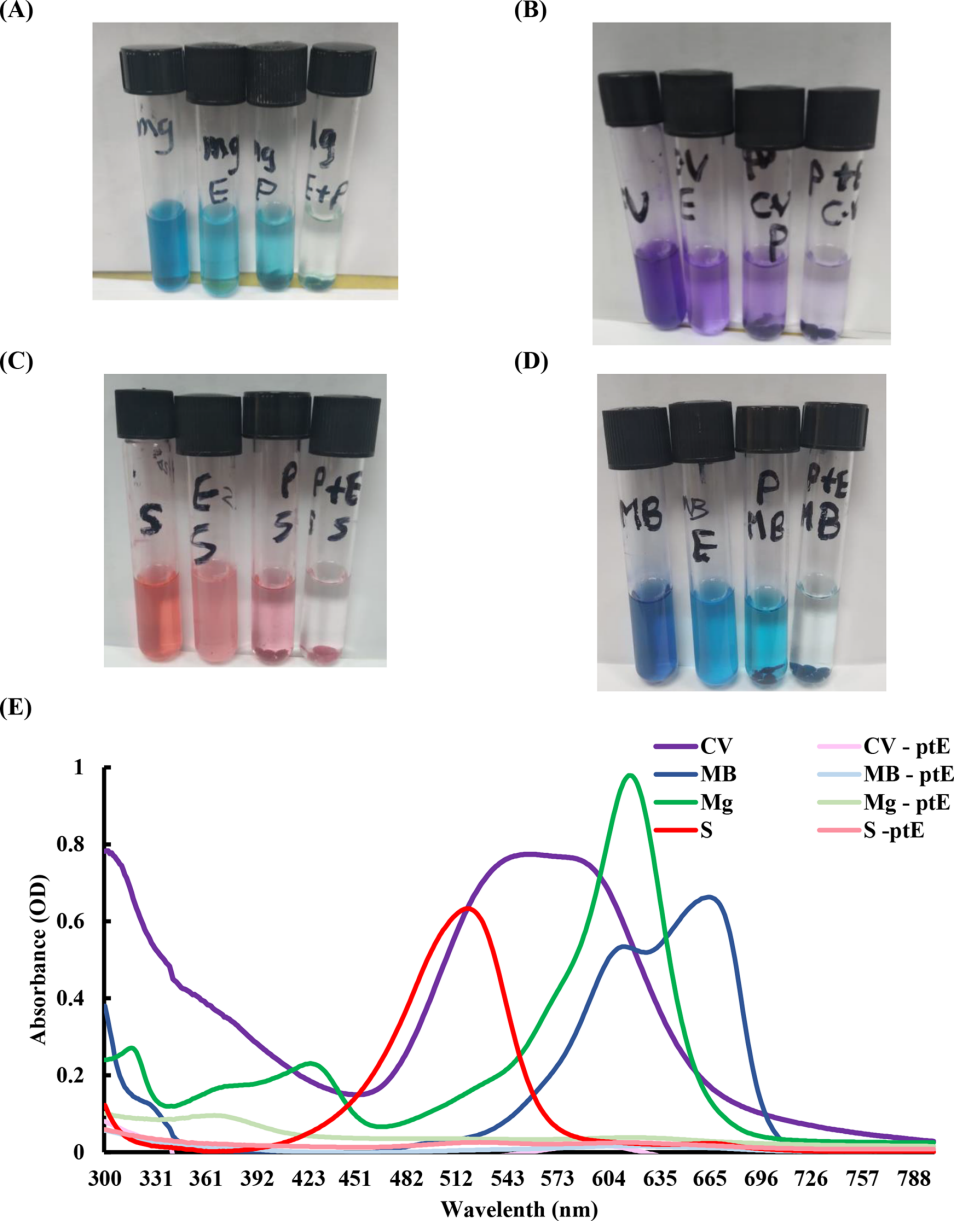
UV/vis spectra of malachite green (MG), crystal violet (CV), methylene blue (MB), and safranin (S) dyes before and after treatment with immobilized recombinant chitinase enzyme

Figure 5 shows the UV-visible absorption spectra of the enzyme-immobilized SA-mRHP beads during the dye removal process. It was observed that the visible region displayed noticeable peaks corresponding to crystal violet (CV) at 620 nm, malachite green (MG) at 521 nm, safranin (S) at 667 nm, and methylene blue (MB) at 570 nm. After the immobilized beads were added, these absorbance peaks vanished and no new peaks appeared (Fig. 6). The dual mechanisms, which include both enzymatic degradation and adsorption onto the beads, may be responsible for the increased dye removal effectiveness of immobilized beads.

### FTIR spectroscopy analyses

**Fig. 7:**
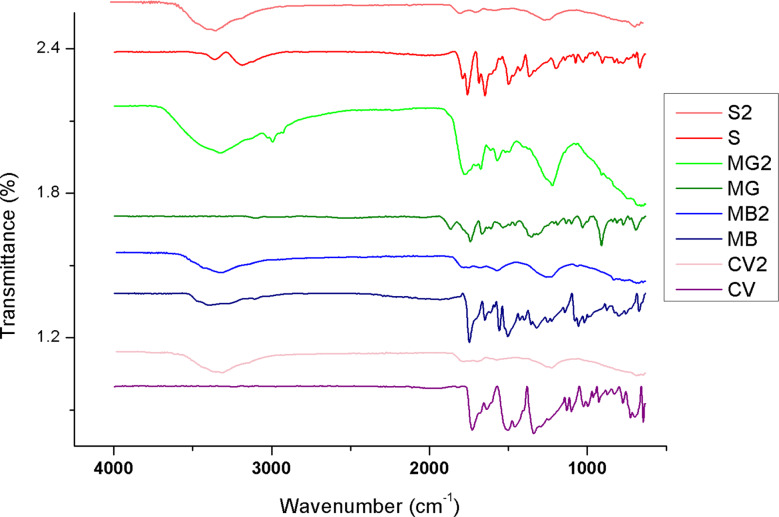
FTIR of Crystal Violet (CV), Methylene Blue (MB), Malachite Green (MG), and Safranin (S) before and after recombinant enzyme addition (CV2, MB2, MG2, and S2)

FTIR analysis was used to determine how the addition of recombinant SmChiA affected various dyes in order to better understand the reaction pathways between the recombinant enzyme and the dyes. Figure 7 displays FTIR spectra of the dyes before and after the enzyme was added. Well-defined peaks of dyes were seen in the spectra (Fig. 7). CV dye exhibited distinct peaks at 1583 cm^− 1^, 1365 cm^− 1^, 1168.90 cm^− 1^, and 1062 cm^− 1^, which corresponded to C = C stretching of the benzene ring, C-H deformation in methyl groups, C-N stretching, and C-H bending vibrations, respectively [69, 70].

The distinctive peaks for MB were found at 1393 cm^− 1^ for CH = N, 1175 cm^− 1^ for the –C–N stretching, 1063 cm^− 1^ for C–S–C, and 1500 cm^− 1^ to 1400 cm^− 1^ for C = C stretching [71]. Accordingly, FTIR spectra of MG showed distinct peaks at 1586 cm^− 1^, 1364 cm^− 1^, and 1172 cm^− 1^, which corresponded to C = C aromatic stretching, C-C aromatic stretching, and C-N stretching, respectively [72]. The spectra of safranin (S) revealed peaks at 1326 cm^− 1^ for aromatic C = N stretching vibrations, 1638 cm^− 1^ and 1608 cm^− 1^ for aromatic ring stretching, and 3320 cm^− 1^ for N-H stretching vibrations [73].

The majority of these distinctive absorption peaks either vanished or became noticeably weaker after 3 d of enzyme addition, demonstrating the destruction of the dye molecules [71, 73–75]. These results are in line with earlier research by Ayed et al., who used *Sphingomonas paucimobilis* to degrade MG dye [76]; and Anburaj et al., who used cyanobacteria *Phormidium* ARKK2 derived from mangroves to achieve MG degradation [74]. Similar FTIR data were found in each of these investigations, suggesting a similar dye molecule breakdown.

### SEM analyses and EDX spectroscopy

**Fig. 8:**
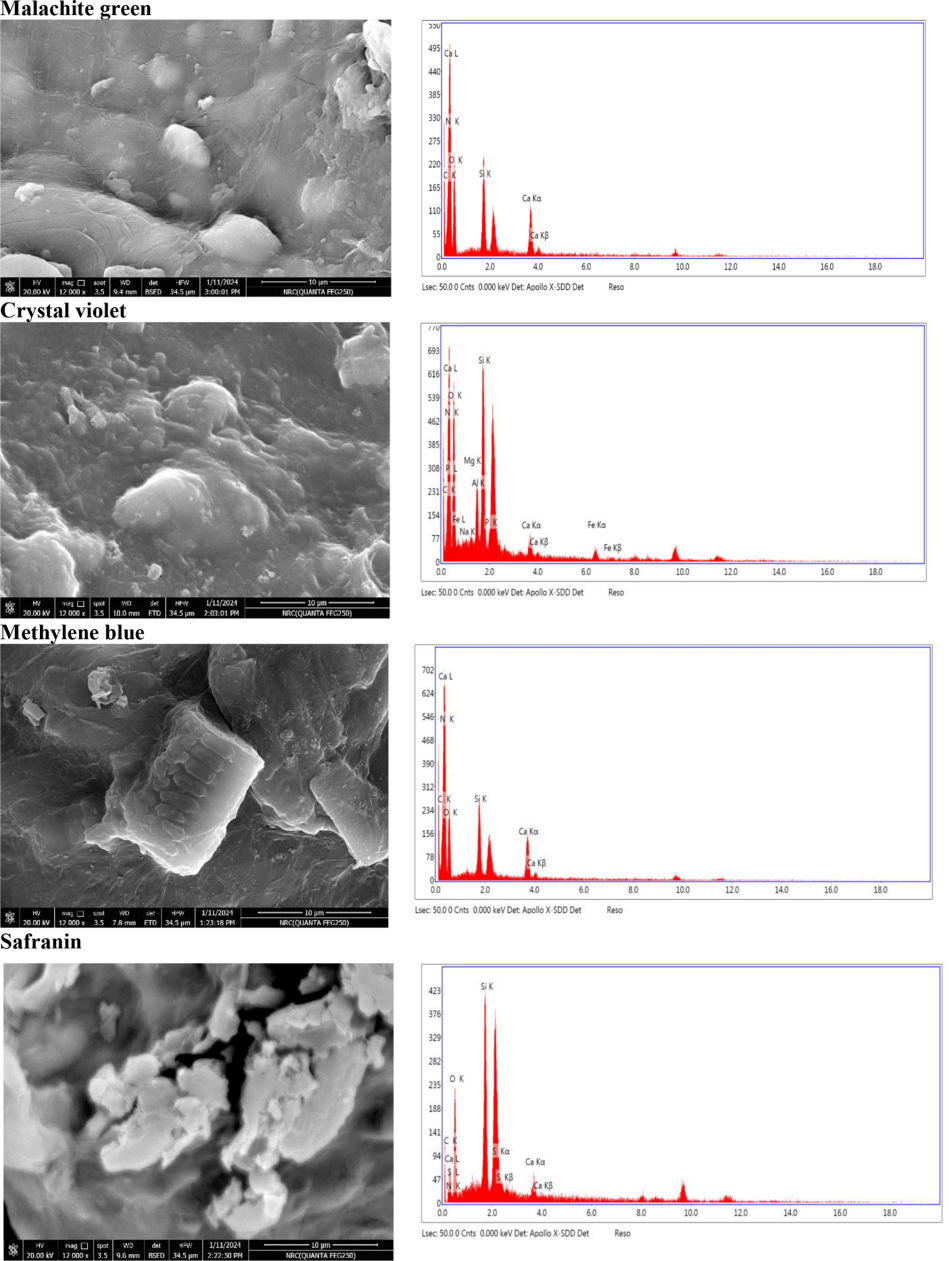
SEM-EDX spectra of the malachite green, crystal violet, methylene blue, and safranin loaded immobilized SmChiA

Following the adsorption of the CV, MG, S, and MB dyes, the SEM and EDX spectra were analyzed for immobilized recombinant SmChiA enzyme (Fig. 8). The elemental analysis of the dye-loaded-enzyme-immobilized adsorbent beads revealed significant amounts of silicon and oxygen. These elements are required for the formation of bonds during the adsorption process. However, the adhesion of the CV, MG, S, and MB dyes to the adsorbent resulted in the appearance of white particle aggregations on the surface. The alterations in the elemental compositions of the adsorbent are depicted by the EDX spectra (Fig. 8). The morphological and elemental changes of immobilized SmChiA with MG, CV, MB, and S dye adsorption indicate physiochemical interactions between the adsorbent and the adsorbate. These findings could increase the efficiency of pollution removal, especially when it comes to dyes.

The atomic weights of C, N, and O was changed, which is definitely due to the adsorption of dye molecule [77]. Confirming that MB and CV dyes were successfully loaded onto the surface of beads, the EDX spectra also revealed the presence of peaks of N and Cl in the EDX spectra of the adsorbed CV (C_25_H_30_ClN_3_) dye and the appearance of peaks of N, S, and Cl in the spectra following MB (C_16_H_18_ClN_3_S) dye adsorption [78]. The presence of N and Cl in the spectra of CV dye adsorbed beads is more evidence that the dye has stuck to the surface of the nanocomposite [79].

## Conclusion

In this study, recombinant SmChiA from *S. marcescens* was used for dye removal for the first time and was chemically immobilized onto SA-mRHP beads. Three different mRHP concentrations (0.5 g, 1.0 g, and 1.5 g relative to SA) were examined. The prepared SA-mRHP beads were characterized using FTIR and SEM, confirming successful enzyme incorporation. Based on BBD optimization, 50% mRHP was identified as the optimal composition for enzyme immobilization. The immobilized SmChiA exhibited broader temperature and pH optima, enhanced thermal stability, and promising dye removal efficiency, particularly for cationic dyes (CV, MG, S, and MB), demonstrating the potential of SA-mRHP beads as an effective carrier for enzyme immobilization in dye removal applications.

## Data Availability

No datasets were generated or analysed during the current study.
